# Investigation of antibacterial properties of polyacrylonitrile fibers modified by new functional groups and silver nanoparticles

**DOI:** 10.55730/1300-0527.3422

**Published:** 2022-04-05

**Authors:** Zeynep OKAY, Meryem KALKAN ERDOĞAN, Başar KARACA, Meral KARAKIŞLA, Mehmet SAÇAK

**Affiliations:** 1 Department of Chemistry, Faculty of Science, Ankara University, Ankara, Turkey; 2 Department of Biology, Faculty of Science, Ankara University, Ankara, Turkey

**Keywords:** Graft copolymerization, poly(acrylonitrile) fiber, Ag particles, antibacterial activity

## Abstract

This work reports the surface modification of polyacrylonitrile (PAN) fibers by graft copolymerization to ensure the decoration of homogenous and dense Ag nanoparticles. Two facile and subsequent modification processes resulted in a PAN fiber composite with an intact fibrous structure, sufficiently conductive for antistatic application and antibacterial activity. In the first step, some chemically attractive monomers and monomer mixtures, such as acrylic acid (AA), AA-itaconic acid (AA-IA), AA-acrylamide (AA-AAm), were introduced to the fiber surface by grafting. The grafting process was evidenced by FTIR, ^1^H-NMR, and SEM techniques. The second step aimed to form a chelate structure by Ag^+^ ions with the coordination centers imparted to the PAN structure, and then, Ag nanoparticles (AgNPs) were decorated on the copolymer fiber surfaces by reducing with the NaBH_4_. The presence, distribution, and changes that occurred after the AgNPs decoration were also monitored by the SEM technique. It was obtained that the AgNPs could not be easily removed from the composites, which presented an appearance as if they were dyed with Ag. It was determined that the composite fibers gained a certain degree of conductivity with the surface resistivity value of 10^9^–10^2^ Ω/cm^2^. The antibacterial activity of the composites against *E. coli* and *S. aureus* was examined by the zone of inhibition test compared to their detergent-washed samples.

## 1. Introduction

PAN fibers are among the most widely used, cost-effective commercial synthetic fibers in the textile industry due to their desired properties, such as high solvent resistance, good insect repellency, and thermal and mechanical stability [1]. However, PAN fiber’s low degree of hydrophilicity and low moisture absorption limit its functionality/usability in various applications. For this reason, to impart new valuable properties to the PAN materials prepared in the form of fiber and membrane, the surface modification route is frequently preferred. For this purpose, many physical or chemical methods are appealed [2–6]. Furthermore, thanks to the improved properties of PAN, it has been suggested that PAN has the potential to be used as an alternative material in the composite technology for manufacturing different materials, ranging from UV protection [7], catalytic investigations [8–11], and sensing [12, 13] to biomedical-chemical applications [14].

The polar nitrile groups in the PAN fiber structure have inspired new approaches to modify the fiber for their specific application. Although the applied physical modifications are rapid and straightforward, the low environmental resistance of the desired property against washing or rubbing effects is found limiting for the selected application field. For this reason, chemical modification methods have been promising in terms of ensuring permanent alteration on the PAN materials. For this purpose, in addition to the strategies that target the hydrolysis and reduction of nitrile groups through enzymatic [15, 16] and chemical [17, 18] processes, the other chemical surface modifications were also carried out for adding desired properties to PAN, such as amidoximation [19–21], plasma processing [22, 23], and photo-induced polymerization [24]. In particular, graft copolymerization of some vinyl monomers, such as acrylic acid, methacrylamide, acrylamide, and hydroxyethyl methacrylate, onto PAN fiber has recently been preferred to impart new properties or to improve its existing ones [2, 4, 5, 25]. In addition, studies reporting the incorporation of metal nanoparticles into fibrous structures are also found noteworthy, in terms of having the advantage of the high surface area of the particles and enabling these fiber composites to be used for different purposes listed above (such as catalysts, sensing, biomedical applications). For example, it was reported that incorporating AgNPs into PAN moiety provides the fibers with excellent catalytic activity, surface-enhanced Raman scattering activity, electrical conductivity, and antimicrobial activity [26]. The uniform and good AgNP immobilization on the PAN surface is also the scope of these studies in this field. At the same time, the modification of surfaces by appropriate functional groups such as NH_2_ and SH enables the homogenous decoration of metal nanoparticles without aggregation through strong chemical interactions on the surface [27–29]. For this purpose, a practical route is suggested to preimmobilize noble metal ions, such as AgNPs, using the chelating effect in the literature [10, 21, 30]. Consequently, after the increase in the use of AgNPs in the production of commercial textile products, studies on the emergence of toxic effects on human health, their stability in the environment, and their low release have been essential issues [31].

This study proposed a modification to prepare composite structures in which AgNPs can be stable, taking advantage of the selected PAN fiber host material properties, such as having a large surface area with a facile modifiable surface and an influential functional group. For this purpose, attempts have been made to increase the functionality of the fiber surface with acrylic acid-based component monomer mixtures. As a result of applying straightforward grafting experiments on the PAN fiber surface in a single step, in addition to the reactive CN functional groups, the COOH and NH_2_ groups, capable of chelating Ag+ ions, were aimed to be chemically introduced to the PAN structure. At the end of these processes, it was aimed to uniformly coat the fiber surface as if dyeing and obtain a relatively resistive texture against harsh conditions such as washing. The opinion that the graft modification method is a suitable method for many metals, metal oxides, metal sulfides, especially in the fibrous structures, has been strengthened by the new data obtained after grafting in this study [9, 32, 33]. To our knowledge, there has been no study reporting both grafting of monomer mixtures and the precipitation of AgNPs and the examination of their antibacterial and electrical properties.

## 2. Materials and methods

### 2.1. Materials

PAN fibers (Mw: 100000, 87% PAN, 13% poly(vinyl acetate)) were obtained from AKSA Co. (Turkey) and used in the experiments after washing in a reference detergent containing aqueous solution at 40 °C for 30 min to clean. Acrylic acid (AA), itaconic acid (IA), and acrylamide (AAm) monomers, silver nitrate (AgNO_3_), and sodium borohydride (NaBH_4_) were purchased from Aldrich and used as received. Benzoyl peroxide (Bz_2_O_2_) (Merck) was crystallized twice from the chloroform/methanol (50/50) (V/V) solvent mixture. The solvents used in all the experiments were of analytical grade and used as received without any further purification. The reference detergent that is conventionally used in the wash fastness tests of the textiles for home laundering was employed in the fiber washing experiment according to the ISO 105-C08:2010 standard.

### 2.2. Methods

#### 2.2.1. Preparation of modified PAN fiber by graft copolymerization

PAN fiber samples (0.300 ± 0.001 g) prepared as small hanks were placed in a 50-mL flask. After impregnating fibers with specific concentrations of monomer or mixtures of aqueous monomer solutions (0.25–2.0 M), the flasks were placed into a thermostat whose temperature was set at 85 °C. The graft copolymerization reactions were initiated by adding a specific concentration of Bz_2_O_2_ solution (3 × 10^−4^ to 3 × 10^−3^ M) in acetone by setting the total solution volume constant at 15 mL and proceeding for 2 h. The copolymer fibers isolated from the polymerization mixture were successively washed by ethyl alcohol and water at the boiling temperature of the solvents under a condenser for 6 h to remove the initiator, monomer, and homopolymer residues. Finally, the graft-modified fibers obtained after drying under vacuum at 50 °C for 12 h were weighed. The amounts of grafted monomers to the PAN structure were determined gravimetrically, considering Equation 1.


(1) $$\text{Grafting yield }(\%)=\frac{{\text{w}}_{\text{g}}-{\text{w}}_{0}}{{\text{w}}_{0}},$$

where w_0_ and w_g_ denote the weights of original and grafted PAN fibers, respectively.

#### 2.2.2. Preparation of graft modified PAN/Ag fiber composite

The graft-modified PAN/Ag composite preparation was carried out in two stages. It was ensured to attach the Ag^+^ ions onto the graft-modified PAN fibers through a chelating effect in the first stage. For this purpose, the modified fibers were stirred in a specific concentration of AgNO_3_ solution at 25 C for 18 h. At the end of the time, the pale-yellow colored Ag^+^-modified fibers were removed from the solution and washed several times with distilled water to remove unattached Ag^+^ ions. In the second step, to complete the reduction of chelating Ag^+^ ions to the AgNPs, an equivalent molar of NaBH_4_ solution was dropwise introduced onto the fibers. After conducting the reduction process at 25 °C for 3 h, the AgNP-decorated fiber composites were isolated from the medium, washed with distilled water, and then dried at 50 °C till reaching constant weight. The Ag contents (%) of modified PAN fibers were calculated gravimetrically, considering the weight differences of the fiber samples before and after the Ag reduction process.

### 2.3. Characterization

The graft modification of the PAN fiber was evidenced by the ATR-FTIR spectra of the samples recorded in the range of 4000–550 cm^−1^ using a PerkinElmer ATR-FTIR instrument. The structural characterization of the samples was also supported by ^1^H-NMR spectra, taken from the Agilent 600 MHz NMR device of the soluble pieces in DMSO-*d_6_*. The surface-resistance measurements of the graft modified PAN fiber/Ag composite samples were performed with the Keithley 6517A Electrometer after the preparation of disks from the samples.

After each surface modification step of the PAN fiber surfaces, the changes in surface morphology and the behavior of the AgNPs on the surface were investigated from the SEM micrographs/EDX spectra of the Au-Pd–deposited samples by Quanta 400F field emission scanning electron microscopy.

### 2.4. Antibacterial activity

The antibacterial activity tests of AgNPs containing untreated and graft-modified PAN fiber composites were investigated against *E. coli* ATCC 35150 (gram-negative) and *S. aureus* ATCC 25923 (gram-positive), through the agar diffusion test, after preparing pellets in 6 mm diameter and 2 mm thickness. The samples were gently incubated in the cultures at 37 °C for 18 h. The antibacterial activity performance was evaluated by taking the averages of the inhibition zone values of two separate samples (in mm). The washing stability of antibacterial activity of the samples was also monitored after applying five cycles of detergent washing, rinsing, and drying processes in 4g/L reference detergent containing aqueous solution (1/50 good to liquor ratio) at 40 °C for 30 min.

## 3. Results and discussion

### 3.1. The graft modification of PAN fiber

In this study, the graft-copolymerization determining conditions, such as polymerization temperature and time, initiator type, and their concentrations, were carried out to increase the number of functional groups on the surface. Thus, as a result of preliminary trials, the effect of the preferred AA monomer concentration and the AA/IA and AA/AAm mixing ratios on the graft yield (%) of the fibers were investigated under the experimental conditions determined as 85 °C and 2 h, respectively [34].

#### 3.1.1. PAN-g-AA fiber

Based on the previous studies on the grafting of vinyl monomers to the PAN fiber [3, 4, 25] in the literature, the effect of Bz_2_O_2_ concentration on the graft yield was preliminarily examined in the 3 × 10^−4^ to 3 × 10^−3^ M concentration range. It was determined in these trials that the graft yield took the highest value at 6 × 10^−4^ M. Since this result is compatible with the grafting studies employing Bz_2_O_2_, 6 × 10^−4^ M was also selected for graft polymerization of AA and its mixtures onto the PAN fiber in this study.

**Figure 1:**
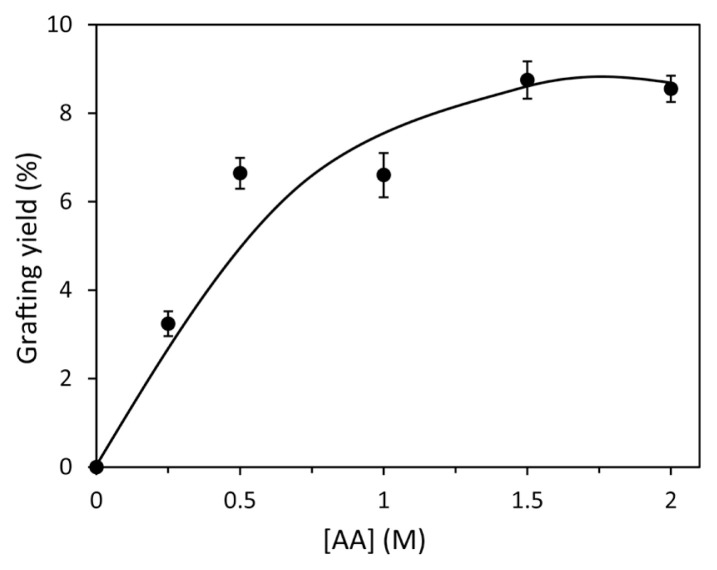
The changes in graft yield values of PAN-*g*-AA fibers with the AA concentration ([Bz_2_O_2_] = 6.0 × 10^−4^ M ).

The graft yield (%) values obtained from the graft copolymerization reactions completed in the 0.25–2.0 M AA concentration range are plotted in Figure 1. As can be seen from the figure, while the graft yield values showed a regular increase with the AA concentration increased up to 1.0 M, no significant change was observed at further concentrations. With the rise of monomer concentration, the number of chains attached to the PAN chain may increase the graft yield to a particular value. However, at higher concentrations, the increase in homopolymer formation in the medium may prevent the diffusion of monomer units to the fiber surface. Thus, the number of grafted chains, in other words, graft yields, may stay unchanged. Similar results were also encountered in the studies reporting the grafting of different vinyl monomers to the PAN fiber [2, 35].

#### 3.1.2. PAN-g-(AA/IA) fiber

**Figure 2:**
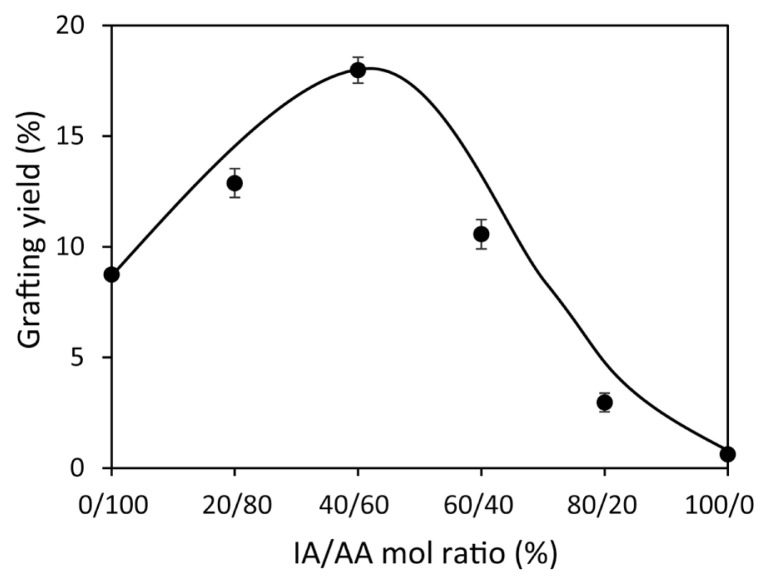
The changes in grafting yield values of the PAN-*g*-(AA/IA) fibers with IA/AA mol ratio ([total monomer]: 1.5 M, ([Bz_2_O_2_]= 6.0 × 10^−4^ M).

It was aimed to enrich the PAN fiber surface in terms of the COOH group by taking advantage of the synergistic effect of the IA monomer in the presence of AA, which is reported to be difficult to graft on synthetic fibers alone [34]. For this purpose, the effect of the AA/IA mole ratio in the monomer mixture on graft yield values of the fibers was investigated. Figure 2 shows the data obtained for PAN-*g*-(AA/IA) fibers prepared in a polymerization medium similar to graft copolymerization conditions with AA. In addition, this graph also compares the grafting yields obtained by keeping the total monomer concentration constant at 1.5 M. Accordingly, it was found that the grafting yield showed a steady increase with decreasing the AA ratio down to 60% ([AA] = 0.9 M) and increasing the IA monomer ratio up to 40% ([IA] = 0.6 M). However, it was also observed that the grafting yield (%) decreased significantly with a further increase of the IA component in the mixture. In the grafting experiment conducted with only IA monomer, it was noted that the grafting remained at the value of 0.6%.

In contrast, in the individual grafting of AA, approximately two times higher graft yield (%) was observed compared to that of grafting of the AA/IA mixture. This result shows AA synergizes in grafting onto the PAN fiber surface, while IA is almost not grafted. Similar results have also been obtained in studies on grafting monomer mixtures to synthetic fibers. It was reported that higher grafting yields could be obtained when some vinyl monomers are employed together compared to their use alone [34, 36–38]. For example, Coşkun et al. reported in a study on the grafting of a mixture of IA and AAm onto PET fiber that the grafting efficiency, which was 3% using IA alone, could be increased up to 76% with the use of AAm in the monomer mixture [34].

#### 3.1.3. PAN-g-(AA/AAm) fiber

In addition to the inclusion of −COOH groups with AA grafting to the PAN fiber structure, the grafting of AAm in the presence of AA was also conducted to ensure incorporating the NH_2_ group, which may play a role in forming the chelate structure with Ag^+^ ions.

**Figure 3:**
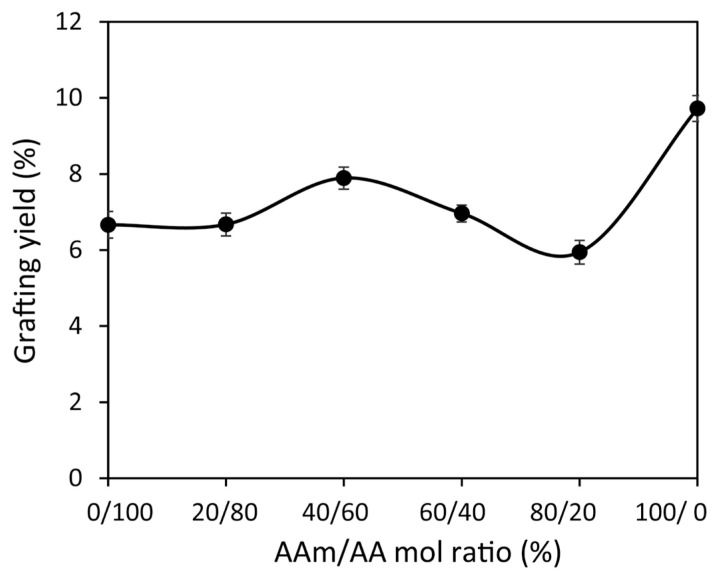
The changes in grafting yield (%) of PAN-*g*-(AA/AAm) fibers with AAm/AA mol ratio ([total monomer]: 1.0 M, ([Bz_2_O_2_] = 6.0 × 10^−4^ M).

During the preparation of PAN-*g*-(AA/AAm) fibers, the changes in grafting yield values were investigated by varying the ratios of the components (%) in the monomer mixture while keeping the total monomer concentration constant at 1.0 M in the mix (Figure 3). As can be seen from the figure, the highest grafting yield (~8%) was obtained when the AA mole ratio was decreased in the mixture to 60%, corresponding to an AAm mole ratio of 40%. It was observed that this maximum grafting value reached was slightly higher than the condition in which only AA monomer was grafted but lower than that obtained with AAm monomer alone. This finding suggests that the synergistic effect observed in the AA-IA mixture was not observed in this mixture.

### 3.2. Graft-modified PAN/Ag fiber composites

In this study, by utilizing the functionalization of PAN fiber surface through grafting technique, it is aimed to precipitate AgNPs in higher yields and smaller particle sizes on the fiber surface, compared to a pure PAN fiber. Thus, it is suggested that the increment of AgNP amount and their penetration to the PAN structure could be ensured. Accordingly, it is expected to provide a composite with a more permanent and higher antibacterial activity.

PAN fibers with a high surface area can carry Ag^+^ ions thanks to −CN groups [39]. The coordination formed between Ag^+^ and −CN is advantageous in the homogeneous distribution of Ag nanoparticles on the surface and minimizing the aggregation of the particles [40].

In addition, new functional groups were added to the PAN fiber by grafting different monomer/monomer mixtures, thus allowing more Ag^+^ ions to form a chelate structure. After the easily applied-reduction process, AgNPs, which may create strong interactions with the fiber surface, could transform PAN fibers into a more advantageous material.

**Figure 4:**
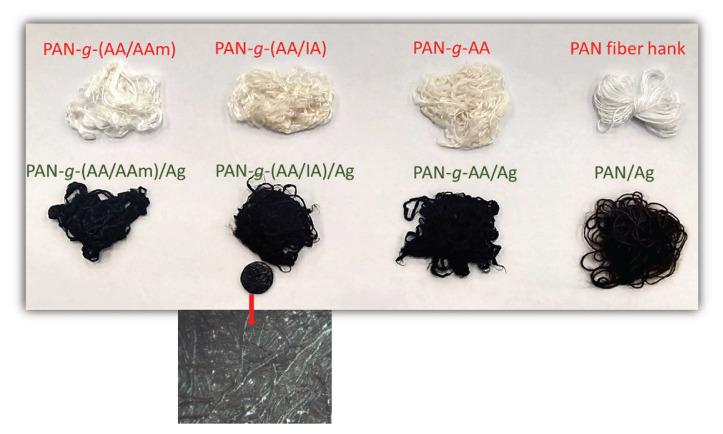
The photographic images of untreated PAN fiber hank, graft-modified PAN fibers, and their AgNP-decorated composites.

In the literature, it is observed that the inability to provide a homogeneous distribution of the particle is a fundamental problem in Ag/polymer film composite studies prepared by the impregnation of Ag^+^ ions and the precipitation of AgNPs [24, 40, 41]. However, in this study, the details of this situation were examined. It was observed that thanks to a particular degree of grafting, homogeneous composite fiber surfaces could be obtained as if painted by Ag black, which can be easily visualized in Figure 4. The optical microscope image given under the photo of PAN-g-(AA/IA)/Ag composite fiber (Figure 4) proves that the surface of pelletized sample evidence the shiny metallic character and homogenous deposition of AgNPs. In addition, it can be seen that no deterioration signs were encountered in the dimensional stability of the fiber after both grafting and the preparation of its composite with Ag.

**Figure 5:**
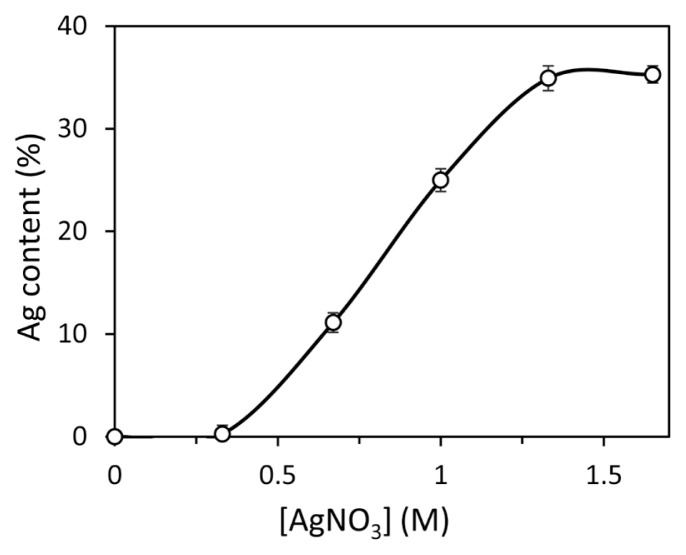
The changes in Ag contents (%) of PAN-*g*-AA fibers with AgNO_3_ concentration (AA grafting yields: ~8%).

In this step, the effect of Ag^+^ solution concentration on the amount of Ag (%) precipitated on the surface of the graft modified fibers was investigated. For this purpose, the fiber samples kept in AgNO_3_ solutions in 5 different concentrations in the range of 0.33–1.67 M were washed with plenty of distilled water until no Ag^+^ was released according to the Cl determination test. Then, the Ag particles were reduced on the fiber with NaBH_4_ aqueous solution at mmol equivalent to the initially selected AgNO_3_ mmol amount. The obtained data were plotted in Figure 5 for the PAN-*g*-AA fiber.

As seen from the figure, the Ag contents (%) of the PAN-*g*-AA/Ag composites steadily increased with AgNO_3_ concentration up to 1.33 M. Over that concentration, it remained almost constant around 35% Ag, depending on the saturation of the fiber surface with Ag^+^ ions. It was also noticed during rinsing that the fibers were degraded when the graft-modified fibers were treated in AgNO_3_ solutions higher than 1.33 M. For this reason, 1.33 M AgNO_3_ solution was also studied in the experiments for the preparation of other PAN-*g*-(AA/IA)/Ag and PAN-*g*-(AA/AAm)/Ag fiber composites.

**Figure 6:**
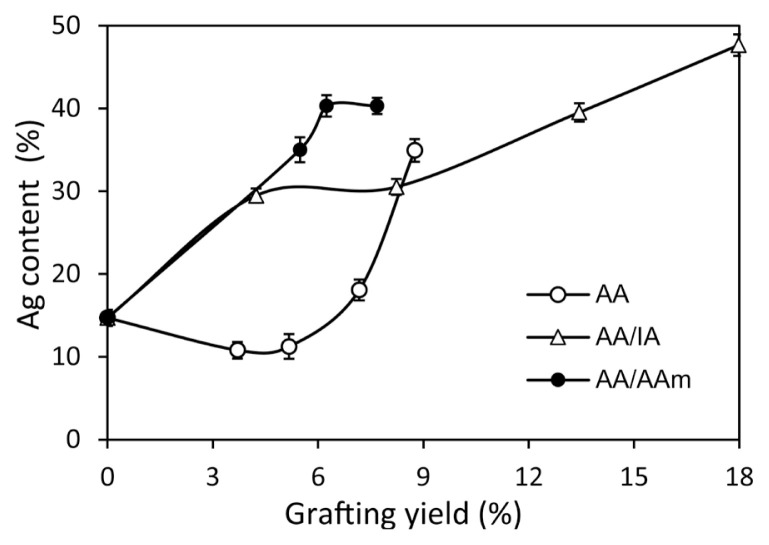
The changes in Ag contents (%) of the graft modified-fibers, PAN-*g*-AA, PAN-*g*-(AA/IA), and PAN-*g*-(AA/IA) with the grafting yields (%) of the fibers ([AgNO_3_] = 1.33 M).

In the next step, the effect of grafting yields on the Ag particles contents (%) of the different graft-modified surfaces was studied, and the results are illustrated in Figure 6.

It was observed that the Ag content (%) precipitated on the fiber surface increased with increasing grafting yields, independent of the differentiation of the grafted units. Notably, the Ag contents (%) took relatively low values in the PAN-*g*-AA fiber series due to the fiber surface’s possible low degree of functionalization by the single -COOH groups. It is also remarkable that PAN-*g*-(AA/AAm) fiber has higher Ag contents (%) at approximately the same grafting yield points compared to other samples, especially PAN-*g*-AA fibers. This situation indicates that the ability of the −NH_2_ group to chelate with Ag^+^ ions may be more effective in the AA/AAm grafting set than AA alone. Nevertheless, since low grafting yields could be obtained in the PAN-*g*-(AA/AAm) fibers, the Ag content values stayed limited and took the highest 40% value at the highest grafting yield point. Since relatively high grafting yields could be achieved in the samples in which the AA/IA mixture was grafted, the most elevated Ag (%) content was recorded as 48% at the highest grafting yield point of 18%. This situation may result from the increment of the chelate forming structures with the increase of the COOH groups added to the structure by the grafting of the AA/IA mixture.

### 3.3. ATR-FTIR spectra

**Figure 7:**
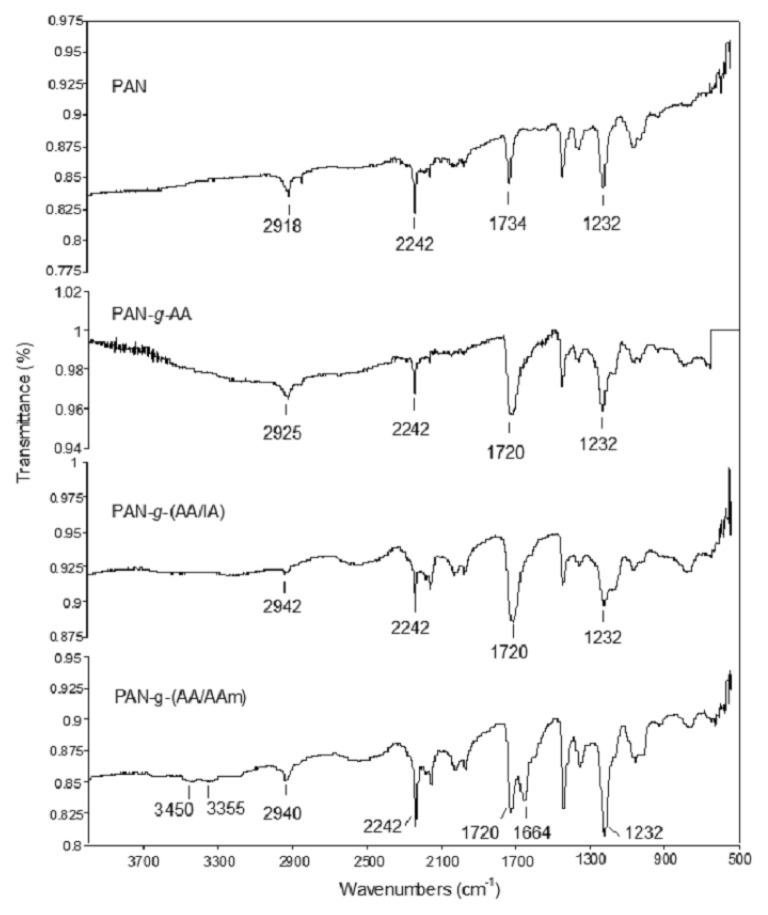
ATR-FTIR spectra of untreated PAN, PAN-*g*-AA (9.5%), PAN-*g*-(AA/IA) (15.6%), and PAN-*g*-(AA/AAm) (7.7%) fibers.

The ATR-FTIR spectra of PAN and graft modified PAN fiber samples are provided in Figure 7.

In the spectrum of PAN fiber, while the bands observed at 2242 cm^−1^ and 2918 cm^−1^ correspond to the characteristic −C≡N and aliphatic methylene ((−CH_2_−) units, the bands at 1734 cm^−1^ and 1232 cm^−1^ arose from the C=O and C-O-C groups present in the poly(vinyl acetate) residue of the fiber [42]. It can be said that the band at 1734 cm^−1^ superimposed with the bands of C=O groups of AA, IA, and AAm units arose at 1720 cm^−1^ after the graft modifications. However, the increased intensity of this band was due to the inclusion of carboxylic acid groups in the structure. This increase is much more pronounced in the PAN-*g*-(AA/IA) fiber spectrum, which may be evidence of the incorporation of IA into the fiber structure by grafting. In addition, in the PAN-*g*-(AA/AAm) spectrum, the second band of C=O stretching vibration of the amide group in AAm units grafted to the fiber appeared at 1664 cm^−1^, while the NH_2_ stretching vibrations were observed as two weak bands at 3355 and 3450 cm^−1^ [43].

The Ag^+^ ions adsorption capability of these functional groups attached through grafting, the FTIR spectra of the samples were also recorded and provided in supplementary info as Figure S1. The assignments of the bonds are also summarized in Table 1. As seen from the table, most of the characteristic bands that were identified previously were preserved after the Ag^+^ ions adsorption with shifting. Some of the strong bands, such as stretching vibrations of C=O groups (1720 cm^−1^) of the grafted fibers, were broadened and split into two bands at 1735 cm^−1^ and ~1700 cm^−1^, which may correspond to the C=O groups of ground PAN and Ag^+^-chelated grafted fibers, respectively [44]. The coordination of metal ions such as Ag^+^ and Zn^+2^ to the carboxyl groups was indicated by the remarkable shifts of the relevant bands in the literature [44, 45]. Therefore, it can be concluded that the PAN-*g*-(AA/IA) and PAN-*g*-(AA/AAm), showing shifts at relatively the highest intensity, were responsible for the high adsorption of Ag^+^ ions to the fibers.

### 3.4. ^1^H-NMR spectra

**Figure 8:**
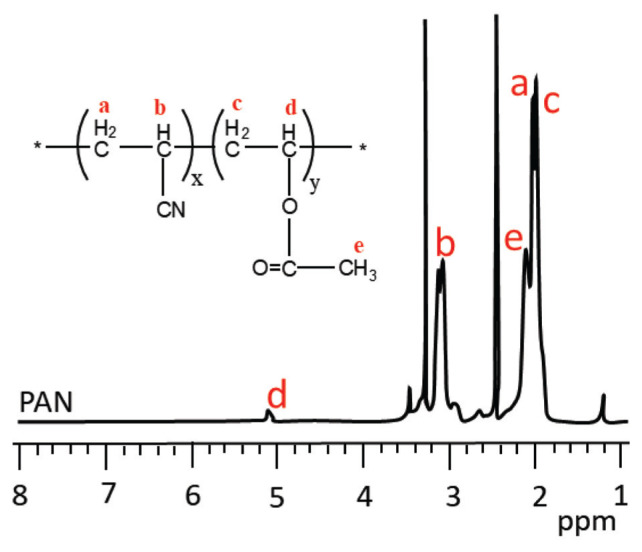
^1^H-NMR spectra of PAN fiber.

**Figure 9:**
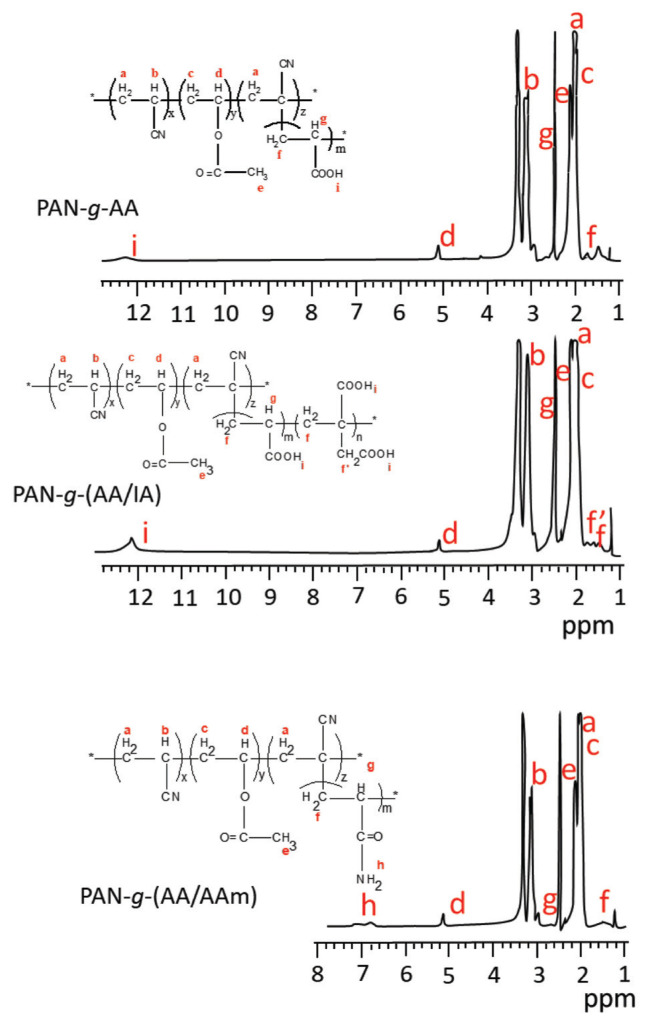
^1^H-NMR spectra of PAN-*g*-AA (9.5%), PAN-*g*-(AA/IA) (15.6%), and PAN-*g*-(AA/AAm) (7.7%) fibers.

For more detailed proof that graft-modified PAN fibers can be prepared, the ^1^H-NMR spectra of the samples were recorded in DMSO-*d*_6_, and the comparative results are successively presented in Figures 8 and 9. In all the spectra, the intense peaks that arose at 3.3 ppm and 2.5 ppm were due to water and DMSO solvents, respectively.

In the spectrum of pure PAN fiber (Figure 8), while the peaks of −CH_2_ and −CH protons are observed at δ = 2.05 ppm (CH_2a_, 2H) and δ = 3–3.2 ppm (CH_b_, 1H), respectively, the proton peaks of poly(vinyl acetate) residue of PAN fiber appeared at δ = 1.9 ppm (CH_2c_, 2H), δ = 5.1 ppm (CH_d_, 1H) and δ = 2.1 ppm (CH_3e_, 3H) [46, 47].

^1^H-NMR spectra of graft-modified PAN fibers are shown in Figure 9. Accordingly, in the spectrum of the PAN-*g*-AA fiber, in addition to the proton peaks of the pure PAN fiber, the peaks of the −CH_2_, −CH, and −COOH protons in the grafted PAA chain structure were obtained at δ = 1.8 ppm (CH_2f_, 2H), δ = 2.05 ppm (CH_2g_, 2H), and δ = 12 ppm (COOH, 1H), respectively [48].

In the spectrum of the PAN-*g*-(AA/IA) fiber, it is evident that the intensities of the −CH_2_ and −COOH proton peaks increase with the IA grafted onto the chain by protecting the chemical shifts of the peaks belonging to the protons of both pure PAN and PAA. However, the protons belonging to similar bonding types, such as −CH_2_ and −COOH, overlap in the spectrum due to them having approximately the same chemical environment. This difference is still noticeable between 1.5 and 1.8 ppm in the spectrum. A similar observation was also encountered in the H-NMR spectra of copolymers of PAN with other vinylic monomers containing acid groups in the literature [49].

In the spectrum of the PAN-*g*-(AA/AAm) fiber, as the possible results of the interaction of the COOH group of PAA with the NH_2_ group of PAAm, low AA/AAm grafting yield, and the predominance of the PAAm structure in the grafted chain, the proton peak of the −COOH group present in the PAA could not be detected. In addition, since the chemical environment of CH_g_ at δ=3 ppm overlapped with the CH_b_ in the pure PAN fiber in the same region, the presence of AA could not be satisfactorily supported by ^1^H-NMR, as obtained by ATR-FTIR spectrum data. In addition, the detection of δ = 7 ppm (NH_2h_, 2H) peak in the spectrum reveals the dominant presence of AAm in the grafted polymer mixture.

To conclude, it could be said that both ^1^H-NMR and ATR-FTIR spectra successfully evidenced the graft modification of the PAN fiber.

### 3.5. SEM micrographs

**Figure 10:**
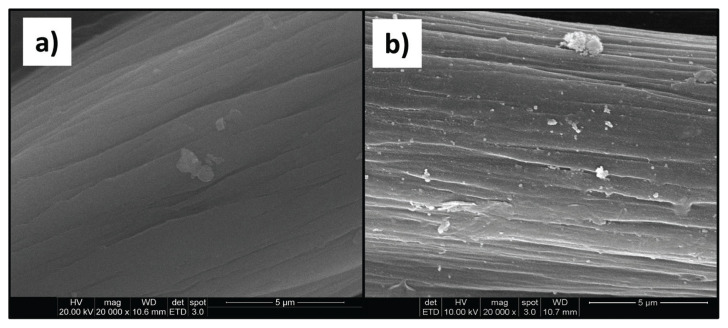
SEM micrographs of a) untreated PAN fiber and b) PAN fiber/Ag composite (14.7% Ag).

It has been known that the stability, size, shape, and morphology of the metal nanoparticles depend on the preparation method. Consequently, SEM micrographs were taken to monitor the effect of the graft chains on the fiber surface morphology and the reduced AgNP sizes, distribution, and aggregation status on the fiber surface after grafting. The results are represented in Figure 10. In the micrograph of the untreated PAN fiber, smooth lines consisting of deep grooves parallel to the longitudinal length of the fiber, which is characteristic of the pure PAN fiber, are noticeable (Figure 10a). In the micrograph of untreated PAN/Ag fiber composite that was prepared compared to the graft-modified PAN/Ag composites, nanosized Ag particles are homogeneously observed by integrating with the surface (Figure 10b). It is also observed that the AgNPs on the surface have a globular structure.

**Figure 11:**
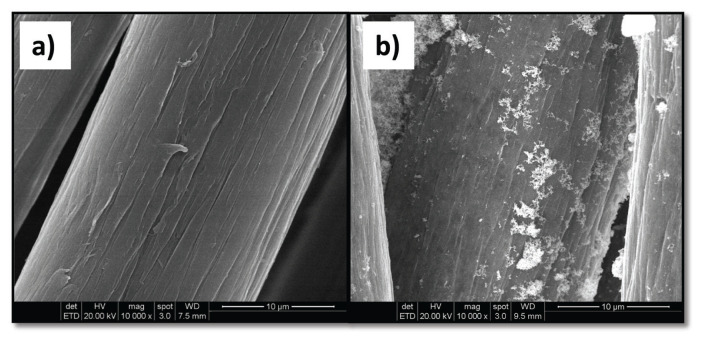
SEM micrographs of a) PAN-*g*-AA fiber (8.8%) and b) PAN-*g*-AA/Ag composite fiber (34.9% Ag).

The micrograph of PAN-*g*-AA fiber (8.5% grafting yield) shows that the grafted PAA chains covered the fiber surface as a smooth film without closing the cavities of the PAN fiber (Figure 11a). Unlike that of the pure PAN/Ag composite, it can be seen from the PAN-*g*-AA/Ag composite micrograph (35% Ag content) that AgNPs capped the surface in spongy-texture, dense, and interconnected spheres on particular regions (Figure 11b).

**Figure 12:**
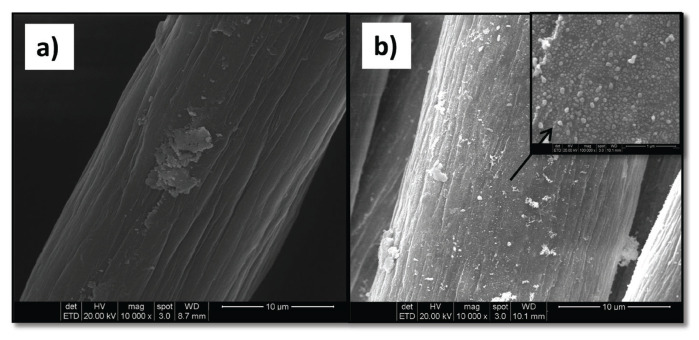
SEM micrographs of a) PAN-*g*-(AA/IA) fiber (18.0%) and b) PAN-*g*-(AA/IA)/Ag composite fiber (47.7% Ag). The inset belongs to the micrograph of PAN-*g*-(AA/IA)/Ag composite at a higher magnification.

In the micrograph of the fiber grafted with a mixture of AA and IA monomers (Figure 12a), a thick layer on the surface can be seen due to the high grafting yield. In the micrograph of the PAN-*g*-(AA/IA)/Ag fiber composite (47.7% Ag content) given in Figure 12b, the presence of relatively dense and nanosized spherical Ag particles that were homogeneously coated without forming agglomerates is easily understood. Additionally, it is seen that the particles are similar to that of the pure PAN/Ag but in slightly larger nano size.

**Figure 13:**
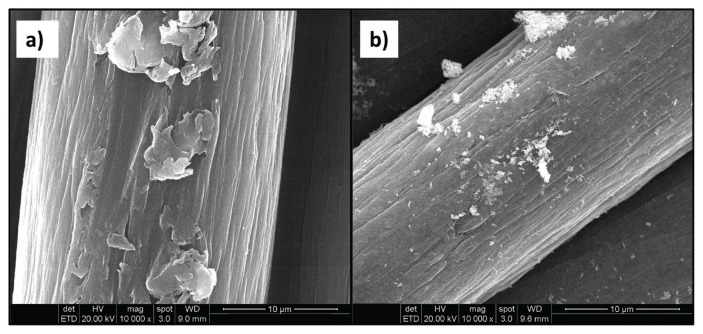
SEM micrographs of a) PAN-*g*-(AA/AAm) fiber (7.7%) and b) PAN-*g*-(AA/AAm)/Ag composite fiber (40.3% Ag).

Since the AA/AAm mixture-grafted fiber has a lower grafting efficiency (6.2%) compared to other graft-modified fibers, the micrograph of the PAN-*g*-(AA/AAm) shows a thin-film coating that can scavenge more easily from the surface (Figure 13a). This morphological difference of the sample from those of other graft-modified structures containing AA can be shown as evidence for the dominance of the AAm in the grafted chain. On the surface of the PAN-*g*-(AA/AAm)/Ag composite, AgNPs show a local distribution that could form large, irregular agglomerates.

### 3.6. Electrical conductivity measurement

Although satisfactorily high Ag content (%) of the composites could be ensured by the graft modification of PAN fiber (approximately ~50%) compared to an untreated PAN (10%), the electrical resistance of the sample could not be directly measured from the surface of an individual fiber. After the fibers were formed into disks, the electrical surface resistances could be measured, and the results are presented in Table 2. According to the results obtained from the SEM micrographs, since relatively the most homogenous and dense decoration of Ag particles could be received on the PAN-*g*-(AA/IA)/Ag composite that would provide electrical conduction in the composite, the measurements were taken from this sample. The table shows that the surface resistivity values decreased by 3 × 10^6^ times with increasing Ag content (%) of the composites from 24% to 47.5%. However, when these values are examined, it can be seen that high electrical conductivity values (here, low surface resistivity) could not be obtained even in the composites containing lower than 40% Ag (%). This situation may arise from the nanoscale reduction of AgNPs on the fiber surface and the inability to provide sufficient particle contact for conductivity. For this reason, it is thought that direct measurements could not be taken from the fiber surface. However, it can be suggested that these resistivity values may be sufficient to provide the antistatic properties to the composites [50, 51].

### 3.7. Antibacterial activity

The antibacterial activities of pure PAN fiber, graft-modified PAN fibers, and their composites containing AgNPs against two different bacteria, including *E. coli* and *S.aureus*, were determined by measuring the inhibition zone diameters. The measurements were also performed after applying five cycles of washing-drying processes to the same samples, and the comparative results are presented in Table 3. As expected from the tests, no response was obtained against the bacteria studied in pure PAN, PAN-*g*-AA, and PAN-*g*-(AA/IA) graft-modified fibers. In the PAN-*g*-(AA/AAm) fiber sample, a 10.5 mm inhibition zone was detected against *S. aureus* thanks to the presence of the −NH_2_ group imparted to the structure by grafting [48]. This observed antibacterial activity of the PAN-*g*-(AA/AAm) fiber is related to the cell wall difference of each bacterium. Due to the lack of an outermost shielding layer such as peptidoglycan, *S. aureus* was reported to be less resistive against the diffusion/susception of external chemicals, which may cause a cell membrane rupture through electrostatic adsorption [52].

When the table is examined, it is seen that graft modified composite fibers containing AgNPs show a certain degree of antibacterial activity against both bacteria. This situation can be interpreted as the release of Ag^+^ ions from the AgNPs of the composites after exposure to the bacterial medium. It can be said that this effect occurs according to the following mechanism given in many studies presented in the literature [53].


$$4\;\text{Ag}+{\text{O}}_{2}+2{\text{H}}_{2}\text{O}↔4{\text{Ag}}^{+}+4{\text{OH}}^{-}$$

As seen in the table, the antibacterial effects of washed-dried composites for five cycles were either higher or unchanged compared to unwashed composites. This situation could be related to the increase in water absorption of polar functional groups added to the surface after the hydrophilic graft modification process by the washing process. Thus, as seen in the mechanism, water molecules absorbed to the surface may have increased the inhibition diameter values of the materials by facilitating the release of Ag^+^ ions from the composites. The appearance of the five cycles of washed PAN-*g*-(AA/IA)/Ag composite from the photograph and optical microscope image taken under 10× magnification were also given in Figure S2.

## 4. Conclusion

This study demonstrates that the surface-modified PAN fiber/Ag composite materials could be developed by the easily controllable graft copolymerization method and the subsequent Ag nanoparticle reduction process. In this way, it has also been shown that graft-modified fibers can be used as versatile template material to decorate AgNPs. It was determined from SEM micrographs that the fiber surface morphology and the distribution of AgNPs on the surface changed depending on the type of monomer/monomer mixtures with different functional groups grafted onto the fibers. The micrographs also revealed that the method applied in the study is suitable for obtaining AgNP-coated fiber matrixes without aggregation. PAN fiber, which is known to be insulating and has low bacterial resistance, can be transformed into a material that can exhibit antistatic and antibacterial behavior with a certain degree of conductivity value. One of the striking results of the study was that the antibacterial behavior of composite materials increased after five washing-drying cycles. It is also thought that PAN fiber/Ag composites, which can be prepared in a few straightforward steps, may be a potential material for catalytic and electronic applications in further studies and washing-resistant antibacterial textile applications.

## Supplementary Information

Figure S1 The ATR-FTIR spectra of the graft modified fibers after the adsorption of Ag^+^ ions.

Figure S2 The photograph and optical microscope image of a PAN-g-(AA/IA)/Ag composite fiber after five washing cycles.

## Figures and Tables

**Table 1:** The ATR-FTIR spectral data for the graft-modified and their Ag^+^ ions adsorbed samples.

Bonding	Corresponding wavenumbers (cm^−1^)					
	PAN-*g*-AA	PAN-*g*-AA/Ag^+^	PAN-*g*-(AA/IA)	PAN-*g*-(AA/IA)/Ag^+^	PAN-*g*-(AA/AAm)	PAN-*g*-(AA/AAm)/Ag^+^
−NH_2_	-	-	-	-	3450(w) and 3355(w)	-
−CH_2_−	2925(m)	2915(w)	2942(m)	2918(w)	2940(m)	2915(w)
−C≡N	2242(s)	2243(w)	2242(s)	2245(m)	2242(s)	2244(m)
C=O	1720(s)	1735(b) and 1705(b)	1720(s)	1735 (b), 1705(b, s), and 1650 (s)	1720(s)	1735 (b) and 1700(b, s)
C=O (amide)	-	-	-	-	1664(s)	1652(s)
C-O and δ(OH)	1232(s)	1290(m) and 1234(w)	1232(s)	1285(s) and 1234(w)	1232(s)	1290(s) and 1231(w)

(s): strong, (m): medium, (w): weak, (b): broadened

**Table 2:** The electrical surface resistivity of the graft modified PAN-*g*-(AA/IA)/Ag composites.

Ag content (%)	Surface resistance (Ω/cm^2^)
24.0	2.0 × 10^9^
30.4	6.6 × 10^5^
39.5	1.2 × 10^3^
47.5	6.7 × 10^2^
42.6*	1.0 × 10^3^*

* After 5 washing cycles

**Table 3:** The inhibition zone diameters of pure PAN fiber, graft modified PAN fibers, and their composites containing Ag particles against *E. coli* and *S. aureus*.

	Inhibition zone diameters (mm)	
Fiber	*E. coli*	*S. aureus*
PAN	-	-
PAN-*g*-AA	-	-
PAN-*g*-(AA/IA)	-	-
PAN-*g*-(AA/AAm)	-	10.50 ± 0.00
PAN-*g*-AA/Ag	8.00 ± 0.71	9.75 ± 0.35
PAN-*g*-AA/Ag (washed)	10.75 ± 0.35	10.75 ± 0.35
PAN-*g*-(AA/IA)/Ag	8.50 ± 0.71	9.25 ± 0.35
PAN-*g*-(AA/IA)/Ag (washed)	12.25 ± 0.35	10.25 ± 0.35
PAN-*g*-(AA/AAm)/Ag	10.25 ± 0.35	11.00 ± 1.41
PAN-*g*-(AA/AAm)/Ag (washed)	10.25 ± 0.35	10.50 ± 0.71
