# Supplementary figures and images for: Investigation of antibacterial properties of polyacrylonitrile fibers modified by new functional groups and silver nanoparticles
Source: Turk J Chem. 2022 Apr 5;46(4):1137–51. doi: 10.55730/1300-0527.3422 (PMC10395732; doi:10.55730/1300-0527.3422)

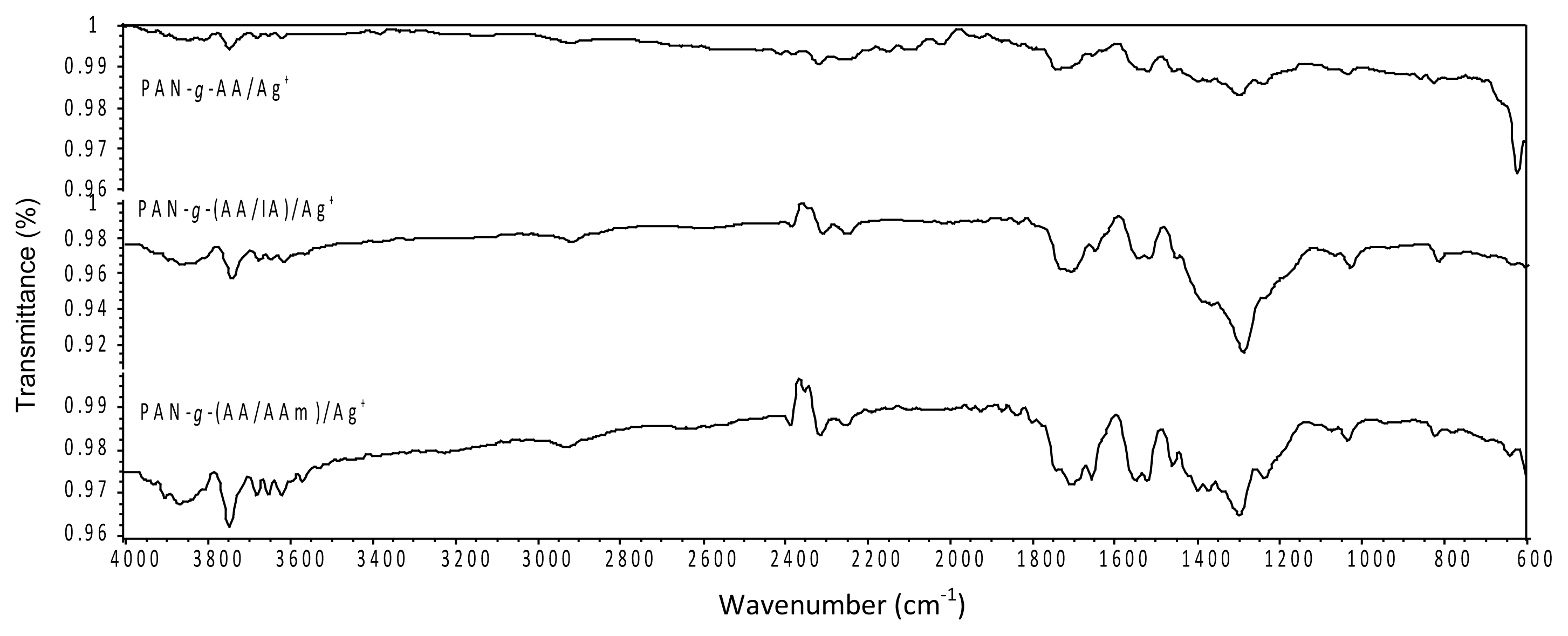

Supplement: Figure S1 — The ATR-FTIR spectra of the graft modified fibers after the adsorption of Ag+ ions. [file turkjchem-46-4-1137s1.tif]

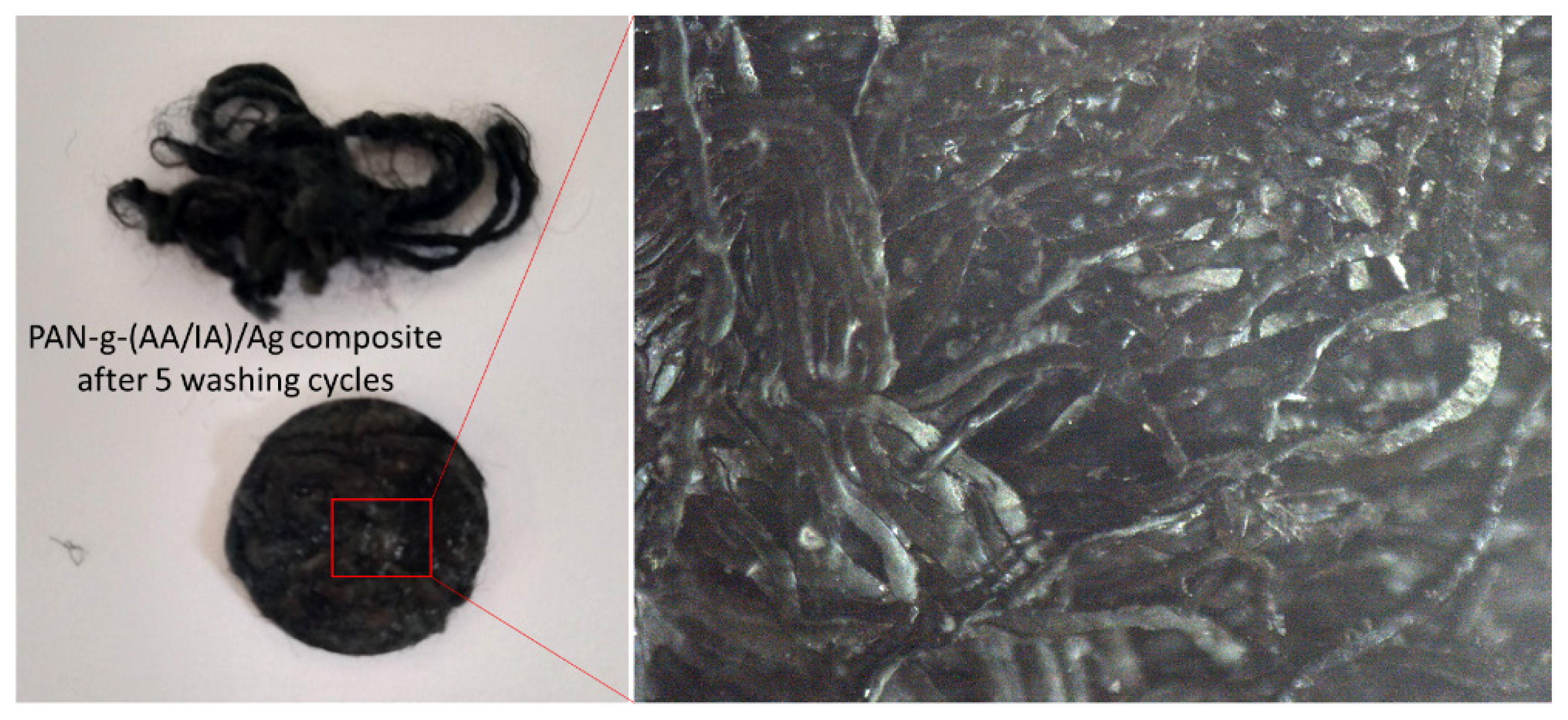

Supplement: Figure S2 — The photograph and optical microscope image of a PAN-g-(AA/IA)/Ag composite fiber after five washing cycles. [file turkjchem-46-4-1137s2.tif]
